# The Energetic Value of Land-Based Foods in Western Hudson Bay and Their Potential to Alleviate Energy Deficits of Starving Adult Male Polar Bears

**DOI:** 10.1371/journal.pone.0128520

**Published:** 2015-06-10

**Authors:** Linda J. Gormezano, Robert F. Rockwell

**Affiliations:** Division of Vertebrate Zoology, American Museum of Natural History, New York, NY, United States of America; University of Aveiro, PORTUGAL

## Abstract

Climate change is predicted to expand the ice-free season in western Hudson Bay and when it grows to 180 days, 28–48% of adult male polar bears are projected to starve unless nutritional deficits can be offset by foods consumed on land. We updated a dynamic energy budget model developed by Molnar et al. to allow influx of additional energy from novel terrestrial foods (lesser snow geese, eggs, caribou) that polar bears currently consume as part of a mixed diet while on land. We calculated the units of each prey, alone and in combination, needed to alleviate these lethal energy deficits under conditions of resting or limited movement (2 km d^-1^) prior to starvation. We further considered the total energy available from each sex and age class of each animal prey over the period they would overlap land-bound polar bears and calculated the maximum number of starving adult males that could be sustained on each food during the ice-free season. Our results suggest that the net energy from land-based food, after subtracting costs of limited movement to obtain it, could eliminate all projected nutritional deficits of starving adult male polar bears and likely other demographic groups as well. The hunting tactics employed, success rates as well as behavior and abundance of each prey will determine the realized energetic values for individual polar bears. Although climate change may cause a phenological mismatch between polar bears and their historical ice-based prey, it may simultaneously yield a new match with certain land-based foods. If polar bears can transition their foraging behavior to effectively exploit these resources, predictions for starvation-related mortality may be overestimated for western Hudson Bay. We also discuss potential complications with stable-carbon isotope studies to evaluate utilization of land-based foods by polar bears including metabolic effects of capture-related stress and consuming a mixed diet.

## Introduction

Climate change is causing the sea ice in arctic regions to melt earlier in spring (e.g., [1,2]), leading to a trophic mismatch between polar bears and their primary spring prey, the pups of ringed seals (*Phoca hispida*) [3]. The bears acquire the majority of their annual energy reserves from hunting seals on the ice, especially during the spring when they capture pups in their snow lairs [4]. In western Hudson Bay, polar bears have historically relied on the energy from hunting these seal pups to sustain them through the ice-free period on land until the ice reforms in fall [5,6]. Assuming that polar bear survival is dependent on access to seals during this critical period, many predict declines in polar bear survival and abundance coincident with the advance of sea ice breakup as polar bears will be forced ashore with smaller fat reserves for longer periods (e.g., [3,7,8]).

Molnár et al. [8] used a mechanistic approach to predict polar bear survival that involved establishing a relationship between physical measures (size and structure) and body composition to determine how energy stores are incrementally depleted as polar bears spend longer periods on land during the ice-free season. The model was parameterized with measurements of captured polar bears in western Hudson Bay and daily maintenance costs that are based on past patterns of average daily weight loss experienced by the bears until they returned to the ice [9,10]. Molnár et al. [8] used the model to predict the proportions of adult males that would starve to death as the ice-free season expands to 180 days, a scenario predicted as ice conditions worsen in response to climate change. The model takes into account somatic maintenance costs and the effects of limited movements (2 km d^-1^) but does not allow for energy influx into the system from consuming additional food on land.

Molnár et al. [8] justified not including a food intake parameter with the assertion that there is no “energetically meaningful” food available for polar bears to eat. They cite Hobson et al. [11] who found that polar bears only utilize fat accumulated from hunting seals prior to coming ashore for energy based on “marine” (as opposed to “terrestrial”) stable carbon isotope signatures in exhaled CO_2_ of polar bears captured on land. Because energy utilization pathways can change under conditions of extreme stress (which polar bears may experience when captured [12]) and since land-based foods, such as geese and marine algae, can possess a marine signature [13,14,15] their assertions may not be valid.

Polar bears are opportunists (e.g., [16,17]) and have been documented consuming various types and combinations of land-based food since the earliest natural history records (e.g., [18,19, 20,21]). While subadults and family groups have been most often observed pursuing terrestrial animal prey [22,23] and eating plants such as berries [16], the spatial distribution of polar bear scats and personal observations (L.J. Gormezano and R.F. Rockwell), suggest that at least some adult males currently consume plants and animals during the ice-free period [21]. In the absence of genetic analyses, the proportion of adult males using land-based resources is not yet known, but it is reasonably assumed that if such foraging occurs and yields some energetic benefit it will increase in frequency (e.g., through social learning) as the needs intensify [24,25].

In this paper, we reconstruct Molnár et al.’s [8] model to predict future survival of adult male polar bears as the ice-free season expands to 180 days, but consider a scenario in which nutritionally stressed bears seek additional terrestrial food when available. Because polar bears have always consumed food on land and such feeding is already incorporated into daily weight loss patterns used to build the original model, we only include novel animal foods (caribou, *Rangifer tarandus*, eggs and Lesser snow geese, hereafter snow geese or LSGO, *Chen caerulescens caerulescens*) that have more recently been identified in the land-based diet [13]. The recent population increases of snow geese and caribou (i.e., 1980s, 1990s) have made them more available to arriving bears, which coincides with the onset of advance in spring ice-breakup [13]. Arriving polar bears now spatially overlap nesting snow goose colonies (see Figure 1 in [26]) as well as local caribou herds which extend across the Cape Churchill Peninsula, south to the Nelson River [13]. Furthermore, as polar bears come ashore earlier they will overlap more of both the incubation periods of snow geese and calving of caribou, potentially creating a new trophic match on land to compensate for the growing mismatch with seals on the earlier disappearing ice.

To evaluate the potential effectiveness of each food toward fulfilling daily energy requirements of adult males projected to starve while on land for 180 days according to Molnár et al. [8], we address these questions:

1. How many individual or combined units of each animal sex and age class (e.g., clutch of eggs, caribou calves) would need to be consumed to prevent starvation in each adult male polar bear?
2. What is the total energy potentially available to polar bears each day from snow geese, eggs and caribou?
3. How many starving adult male polar bears could be supported by each food source?

We discuss the limitations of our derived energy calculations in light of the absence of rigorous data on certain aspects of polar bear foraging behavior such as locomotive costs associated with different foraging techniques.

## Methods

### Polar bear energy budget during the ice-free season

We used a 2 component, dynamic energy budget model [27] developed by Molnár et al. [8] to track daily energy expenditures and potential deficits that polar bears could accrue while on land as the ice-free season expands. Daily expenditures were presented as the change in storage energy utilizations for somatic maintenance and movement over time. Parameters such as metabolic rate and fat reserves were modeled with straight-line body length and total body mass of different sex and age classes of polar bears captured on land in western and southern Hudson Bay. Based on these relationships, daily estimates of structural volume and energy stores were generated and used to predict critical thresholds beyond which starvation occurred [8].

Application of this model is limited to adult male polar bears (≥ 7 years old) during the ice-free season, so other draws on storage energy, such as thermoregulation, structural growth and reproduction are not accounted for. Because Molnár et al. [8] assume that there is no influx of energy from foods consumed during this period, the general model (Eq (2) in [8] changes solely as a function of daily expenditures, including somatic maintenance and movement:

$$\begin{array}{l} \frac{dE}{dt}\;=\;\underset{}{\underset{︸}{-mLBM}}\;\underset{}{\underset{︸}{-(aM^{b}+cM^{d}v)}}\\ \text{ Somatic maintenance Movement} \end{array}$$ (1)

where somatic maintenance is assumed to be proportional to the costs associated with maintenance of lean body mass (*LBM*) and the metabolic rate (*m*) is the energy required to maintain a unit mass of lean tissue [10]. Movement costs were derived from an allometric equation describing how costs change as a function of total body mass, *M* [10]. The first component, postural costs, (*aM* ^*b*^) describes metabolic costs associated with standing and the second, *cM* ^*b*^ *v*, describes how energy consumption increases linearly as a function of velocity, *v* [28,29].

As explained by Molnar et al. [10], Eq (1) can further be expanded and parameterized using the body composition model:

$$\begin{array}{l} \frac{dE}{dt}=\underset{}{\underset{︸}{-m(\alpha^{-1}(1-\varphi )E+\rho_{STR}kL^{3})}}\\ \text{ Somatic maintenance}\\ \;\underset{}{\underset{︸}{-(a{\left(a^{-1}E+\rho_{STR}kL^{3}\right)}^{b}+c{\left(\alpha^{-1}E+\rho_{STR}kL^{3}\right)}^{d}v}}\\ \text{ Movement} \end{array}$$ (2)

Where *α* represents the energy density of storage, *φ* is the proportion of storage mass that is fat and *ρ*_*STR*_ *k* is a constant to estimate structural mass from straight-line body length, *L*. Storage energy, *E*, can be expressed as a function of total body mass and straight-line body length (Eq (1) in [10]):

$$E=\alpha (M=\rho_{STR}kL^{3})$$ (3)

Following [10], body composition and maintenance parameters were estimated as *m* = 0.089 MJ kg^-1^ d^-1^, *α* = 19.50 MJ km^-1^, *φ* = 0.439, *ρ*_*STR*_ *k* = 14.94 kg m^-3^ and movement parameters were estimated as *a* = 0, *c* = 0.0214 MJ km^-1^ and *d* = 0.684 [30]. Parameter *b* is not reported but we assume this is because *a* = 0, so postural costs must equal zero, regardless of the value of *b*.

Most adult males are reported to be inactive on land during the ice-free season [31], however, movement rates of approximately 2 km d^-1^ have been reported [32] in western Hudson Bay. Molnár et al. [8] consider both scenarios, where *v* = 0 (i.e., somatic maintenance only) and *v* = 2 km d^-1^ for calculations of energy costs. Also, they observed little variation in straight-line body length among the adult males sampled, so a mean length (*L* = 2.34 m) was used in all calculations. With initial energy stores, *E*_*0*_, the time to death by starvation was computed by numerically integrating Eq (2) and solving for time *T* when *E(T)* = 0 [8]. Two ice-free season threshold lengths were used to compare starvation rates among adult males during times of contrasting climate conditions: 120 days, typical of the 1980s, and 180 days to represent potential future conditions as warming trends progress. Using measurements for 97 adult male polar bears captured in 1989–1996, and assuming those sampled bears were representative of all adult males in the western Hudson Bay population, Molnár et al. [8] estimated that approximately 3% died of starvation at the end of a 120-day period if resting and 6% if walking 2 km d^-1^. As that period expands to 180 days, 28% and 48% would die of starvation if resting or walking, respectively. For sake of reference, adult males comprise approximately 25% (234 polar bears) of the western Hudson Bay population (*N* = 935 in 2004) based on proportions captured during darting operations once surveys were expanded to include all age and sex classes [33].

To reproduce their results, we computed energy density values (*E/LBM)* for sequential mass values (in 1 kg intervals) using Eqs (2) and (3):

$$\frac{E}{LBM}=\frac{\alpha (M-\rho_{STR}kL^{3})}{\left(\alpha^{-1}(1-\varphi )*\alpha (M-\rho_{STR}kL^{3})+\rho_{STR}kL^{3}\right)}$$ (4)

and matched the mass values associated with the energy densities for 97 adult male polar bears extracted from Figure 3 in Molnár et al. [8]. Using discrete numerical calculations, we reproduced the daily energy usages for each of the 97 adult male polar bears under scenarios of resting or walking and for 180 days. Under scenarios of resting and walking, we iteratively calculated the daily energy required to prevent starvation by adding the somatic maintenance and movement costs (*v* = 2) for the mass that the bear was on the day before energy stores reached zero. Movement costs were added to the new daily energy requirements regardless of whether these bears had been “moving” prior to starving because movement would be necessary to obtain food from that point forward. The daily energy requirements were summed across all remaining days within the 180-day span for each starving bear (hereafter total energy deficit) and ranked by total value. These data are illustrated by listing the number of starving polar bears with total energy deficits in sequential 50,000 kcal groupings.

### Food availability during a 180-day ice-free season

Although sea ice concentration and extent have delayed freeze-up in parts of Hudson Bay [2], expansion of the ice-free season thus far has mainly been attributed to earlier breakup [1,7]. For this reason, we only consider an annual advance in spring sea-ice breakup to predict when Hudson Bay would be ice-free for 180 days and thus when polar bears would be forced ashore for that duration. We calculated this date following Rockwell and Gormezano [34] by linearly projecting a 0.72 d yr^-1^ advance from the average breakup date observed in the 1980s (1980–1989), when an ice-free period of 120 days was typical [8]. The year when this annual advance resulted in a 60-day expansion of the ice-free period (180–120 = 60) was 2068.

To estimate snow goose arrival, breeding and molt during the 180-day ice-free season, we projected the mean hatch date in 2068 based on a 0.16 d^-1^ yr^-1^ advance from 2008 (21 June) [34]. Caribou are cued to initiate spring migration to the calving grounds based on day length and studies in other caribou populations indicate that calving date has advanced little in response to climate change [35]. We, therefore, used the 2013 estimated calving date, 1 June, for energy calculations in 2068. For sake of simplicity, we used 2013 estimates of population size for LSGO (71,068 nesting pairs) and caribou (minimum count of 3000) for energy calculations in 2068.

### Energy compensation to starving polar bears

Translating energy available into energy required to prevent starvation is difficult in a species for which there is little information available on actual terrestrial foraging behaviors or the energetic costs and dynamics associated with those behaviors. In dealing with this uncertainty, we make an initial attempt at integrating the energy available with energy needed by examining maximum potentials and then computing the foraging efficiency that would be required for the translation.

We tabulated the total energy that would be available from each food in 2068 as the ice-free season expands to 180 days. We then compared these energy estimates to different deficit levels that are projected for polar bears that will be susceptible to starvation (28% of resting and 48% of walking bears) according to Molnár et al.’s [8] model. Total energy deficits for each starving bear were ranked into 5^th^ (highest energy requirements), 25^th^, 50^th^, 75^th^ and 95^th^ (lowest energy requirements) percentiles with each computed from the average of all energy values falling within 2.5 percentage points (above or below) each of the aforementioned percentiles. We then calculated how many units of each food item (e.g., clutches of eggs, individual animals) could maximally compensate for the total energy deficits of starving bears in each of the 5 energy condition percentiles for bears that were either resting or walking 2 km d^-1^ prior to starvation assuming only the added 2 km d^-1^ movement costs (and no additional energetic cost) to procure each food item. Also, because polar bears often consume different foods together [21], we provide an example of potential combinations of foods, based on patterns observed in polar bear scat, that together compensate for total energy deficits in each percentile for resting or walking bears.

Adult male polar bears have been observed pursuing and consuming each of the food items discussed, which suggests that the behavior could become widespread through social learning and energetic need [22,25]. For this reason, we also modeled the total number of starving adult male polar bears that could be supported by each of the food items (eggs, LSGO, caribou) as the ice-free season expanded to 180 days. For each day polar bears overlapped a food source, the total available energy from each food (eggs, goslings, pre-hatch adult females, flightless adults, calves, yearlings, cows, bulls) was tabulated. For this analysis, we only considered an “average” year for gosling survival and used the minimum estimate of 3000 caribou in the Churchill area (see “Computing the potential caloric values of land-based foods”). Using Molnár et al.’s [8] proportions of 97 adult male polar bears that would starve in 180 days, we calculated the energy needed for somatic maintenance and movement costs at 2 km d^-1^ (whether or not they had been walking previously) at the mass the day before they would starve (*E(t)* = 0). Because most of the starving bears depleted their energy reserves at approximately the same mass ($\bar{x}$ = 191.93 kg, *SD* = 0.2512) the daily energy requirements (including both somatic and movement costs) did not differ much between individuals so we used the mean value (4450.28 kcal d^-1^, *SD* = 5.37) in calculations. We divided the total energy value of each food item, summed across days, by the mean daily energy deficit (4450.28 kcal) multiplied by the maximum number of days that a starving polar bear would need daily energy supplementation (122 days) to obtain the minimum number of adult male polar bears coming ashore susceptible to starvation that could be supported by each food. This is a conservative estimate of supported bears because individual bears depleted their energy reserves at various points within the 180-day span depending on their arrival mass. 122 days represents the longest period over which males in the worst condition would need to supplementation (days 59 through 180); all others would require food for shorter periods of time. In accordance with Molnar et al. [8], we use the same distribution of arrival masses for calculations of adult male survival as were observed during the 1980s and 1990s. The lack of procurement costs, other than those for movement 2 km d^-1^, in calculations of daily energy requirements may lead to overestimation of the number of bears supported by each food item so these estimates should be considered the maximum limits.

Recreation of energy profiles for individual polar bears and other computations were completed using R 3.0.1 [36].

### Computing the potential caloric values of land-based foods

#### Snow Geese

In 2006, the nesting population of snow geese on the Cape Churchill Peninsula (CCP) was estimated to be 48,885 pairs [34]. In response to management actions taken to control the Mid-continent Population of snow geese, adult survival had been reduced since 1996 and the population was thought to be nearly stationary [37]. After 2006, however, adult survival increased [38] and the population has again been growing at its pre-management rate of λ = 1.05 to 1.06 (R.F. Rockwell, unpublished data). Because a complete inventory of the CCP snow geese is not scheduled until 2016, we estimated the 2013 population size by projecting the 2006 value forward with a discrete time geometric growth using the midpoint of the population growth estimate as 48,855 × 1.055^7^ = 71,068 pairs of nesting geese.

We used the fat and protein content of newly laid snow goose eggs estimated by Badzinski et al. [39] and described the changes in caloric worth over the 24-day incubation period from patterns of decline as the yolk content is consumed by the embryo [40,41]. We projected a peak hatch date of 20 June for 2013 based on 0.16 day per year advance since 2006 (21 June) described in Rockwell and Gormezano [34]. The actual hatch date will vary over a span of 7 days each year due to asynchronous nest initiation [34]. Using the peak (or mean) hatch date will result in a slightly different projected overlap with polar bear arrival from what was reported earlier based on annual advance rates [34]. Assuming a 1:1 sex ratio among adults [42] and all females bred, we estimated energy values for 71,068 clutches and 284,272 eggs, using a modal clutch size of 4. We calculated values for partial clutches for the 3 days after laying was initiated until day 4 when most clutches were complete (i.e., contained 4 eggs) and assumed that both eggs and adult females would be vulnerable to predation during laying and incubation. A daily nest survival rate was computed based on an overall nesting success of 91.5% over the 24-day incubation period (0.9962 = 0.915^(1/23 days)^) [26].

Post-hatch gosling survival varies annually, depending, in great part, on the degree to which hatch coincides with peak emergence of wetland grasses (e.g., *Puccinellia phryganodes*) that goslings forage upon [42]. Years of closer match between hatch and peak emergence of graminoids (hereafter “good years”) results in higher survival rates 30 days after hatch (e.g., 2013 *s* = 0.795, computed from the decline in the proportion of goslings between hatch and banding operations 30 days later when the proportion of goslings is again estimated). Years when hatch precedes graminoid emergence (hereafter “bad” years) result in lower survival during the same period (e.g., 2007, *s* = 0.525). The number of goslings on day 1 (260,109) was computed from the proportion of successful nests multiplied by 4 (modal clutch size). Gosling numbers from day 2 to 30 were computed using daily survival estimates for good (0.9921 = 0.795^(1/29 days)^), bad (0.9807 = 0.525^(1/29 days)^) and an average year (0.9864 = 0.660^(1/29 days)^), using the midpoint of good and bad.

Fat and protein values were available for neonates [43], however only body mass and protein measures could be obtained for growing goslings (at days 31 and 43) from Akimiski Island, Nunavut [44], where snow geese are generally smaller than those nesting further north on the CCP (R.F. Rockwell pers. obs.). To establish general relationships describing increases of both protein and body mass, based on 3 measures (days 1, 31 and 43), we calculated the daily average geometric growth rates between measurements using the following equation:

$$Daily\;Growth\;Rate\text{ = }{\left(m_{i+1}/m_{i}\right)}^{1/(t_{i+1}-t_{i})}$$ (5)

where *m* is the measured content (e.g., mass, protein) in kilograms and *t* is time in days between the measurements. Between days 1 and 31 daily increases in protein and mass were 1.1164 and 1.0988 grams, respectively, and between days 32 and 43, growth slowed to 1.0175 and 1.0128 grams of protein and mass, respectively.

To relate the proportion of protein to mass of the goslings observed on Akimiski Island to the larger ones on the CCP, we first used regression to establish a general relationship of how body mass of CCP goslings changes through the growth period. Using body mass values of neonates from the McConnell River in Nunavut (i.e., similar mass to CCP neonates) [43] and those from the CCP from days 23 to 50 (R.F. Rockwell, unpublished data), we fit a power function to describe changes in body mass with time (y = 85.479x^0.7766^; R^2^ = 0.99). We then multiplied ratios of protein to body mass calculated from the Akimiski Island gosling data [44] to the masses of goslings in CCP to estimate daily protein content.

To estimate fat content of goslings, we used a lipid index model from Table 4b in Aubin et al. [45] that describes how fat reserves decrease with gosling age. We scaled the index units to known lipid values (in grams) for neonates (i.e., day 1) [39,46] and fit the data to a power function (y = 0.562x^-0.992^; R^2^ = 0.74) that predicts daily fat content and suggests a drastic drop after day 3 when the remaining yolk is exhausted by the gosling. Grams of fat and protein were converted to gross energy using standard coefficients of 9.39 and 4.30 kcal g^-1^, respectively [47,48]. We further scaled these by the digestibilities of fat and protein for polar bears (0.97 and 0.84, respectively) provided by Best [49].

Both adult males and females are present during incubation, however, females of both snow geese and common eiders (*Somateria mollissima*) have been observed being attacked by polar bears while guarding their nests [50]. During these attacks the females are stalked slowly then rushed by polar bears [50] suggesting that females are vulnerable to predation during this period even though capable of flight. For this reason, we include the caloric value of adult females (not males) from the initiation of laying through hatch using fat and protein values from Ankney and McInnes [51]. We fit a power function (y = 2E+19x^-7.31^; R^2^ = 0.99) to the calculated available energy (kcal) modeled with time for the laying, early and late incubation periods and predicted daily energy values from day 1 of incubation through hatch for 35,534 females.

Approximately 18 days after hatch, adults begin molting their flight feathers [52,53] and both sexes are vulnerable to predation (e.g., [22,54]). We calculated the available energy (kcal) from protein reserves (fat content is negligible) of both adult males and female during the post-hatch, early and late molt periods [51,55]. A linear regression model was fit to the average available energy of males and females with time (y = 2E+06x^-1.491^; R^2^ = 0.63) and used to predict energy values from the beginning of molt (2 July) to flying, approximately 4 weeks later (2 August). Gross energy from protein was scaled by Best’s [49] estimate of digestibility (0.84).

#### Caribou

Unlike the case for snow geese, there is no long-term database available for the caribou of the CCP. In generating our estimates of available calories, we have relied on all information there is on this herd and information from studies of mostly nearby herds. Rigorous population surveys are lacking for the Churchill caribou herd, however recent counts (in 2005 and 2012) suggest a stable minimum population size of approximately 3000 animals (R. Brook and C. Elliott pers. comm., [56,57]). Using this value as a lower limit, we consider caloric values from a total of 3000, 4000 and 5000 individuals (D. Hedman, pers. comm., [56,57]) to reflect the uncertainty surrounding population size. The number of adults was estimated based on a sex ratio of 0.85:1, males to females [58].

Calves were most often first sighted on or around 1 June in the CCP (R.F. Rockwell, pers. comm.), so this date was chosen as day 1 to determine calf composition based on daily growth with age. This date seems reasonable since it occurs midway between peak calving in the Pen Islands herd to the south (17–28 May) [59,60] and the Qamanirjuaq herd to the north (5–15 June) [61]. Although data from collared females in the Churchill herd indicate that adult females typically migrate toward the coast in April (V. Trim, pers. comm.), we considered all age and sex classes of caribou to be vulnerable to predation by polar bears from the onset of calving (1 June) until they leave the coast by 15 October (total days = 137 days) (V. Trim, pers. comm.)

Calf survival was based on observed proportions of calves in the population and estimates of mortality during different times of the year. We used an estimate of 21.1% calves during the post-calving period (~ 1 July) based on the average of counts from 2008 (23.1%) and 2009 (16.1%) in the Pen Islands Herd [60]. We estimated calf mortality (28.6%) from birth to the post-calving period (1 July), based on average mortality estimates for the Porcupine herd in Alaska during the first month after birth (1983–1985) [62]. Using the proportion of calves present in the population on 1 July (21.1%) and calculating the average daily survival from 1 June (birth) to 1 July (0.9888 = 0.714^(1/30 days)^), we estimated that neonate calves (prior to mortality) comprised 29.6% of the population. We used an estimate of over-winter calf survival (14.7%) based on average calf to adult ratios (17.2:100) from late winter surveys of the Churchill herd conducted in 2012 and 2013 (V. Trim, pers. comm.) to calculate the survival rate (0.6967) from 1 July to 1 March based on the average change in proportion of calves in the population during this period. The average daily survival rate during this period was calculated in a similar fashion as above (0.9985 = 0.6967^(1/242 days)^).

We also used the proportion of calves in the population that survived the winter (14.7%) to estimate the proportion of yearlings available during summer. Given that 12–15% recruitment is generally considered to be the threshold for population stability in caribou populations [63] and the Churchill herd is considered relatively stable [57], an estimate of 14.7% is not unreasonable.

The fat and protein content (kg) of adult females and calves at different stages of growth were taken from Gerhart et al. [64] based on data from the Central Arctic and Porcupine herds. Gerhart et al. [64] developed a series of equations to predict fat and protein content of caribou from measurements of total body mass (kg). We used standard coefficients, 4.30 and 9.39 kcal g^-1^, to convert predicted fat and protein, respectively, to gross energy. Available energy was calculated using digestibilities of fat and protein (0.97 and 0.84, respectively) provided by Best [49]. Energy values for adult males and yearlings were predicted using these relationships from body mass (kg) values of Svalbard reindeer (for adult males) [65] and Svalbard reindeer and caribou (for yearlings) [65,66] at various times of the year.

Daily changes in fat and protein composition for adults, yearlings and calves were calculated using Eq (5). Daily growth rates of fat and protein in calves were calculated based on measurements obtained for 1 Jun, 27 Jun, 23 Oct., 11 Sep. 13 Oct., corresponding to days 1–134 from birth [64]. Measurements for adult females were obtained for 7 May, 7 Jul., and 3 Oct. [64]. We obtained monthly estimates of body mass for adult males and yearlings and estimated daily growth rates from the mid-point of each month, including May, Jun., Aug., Oct. (and Nov. for adult males only) [65,66].

## Results

**Fig 1:**
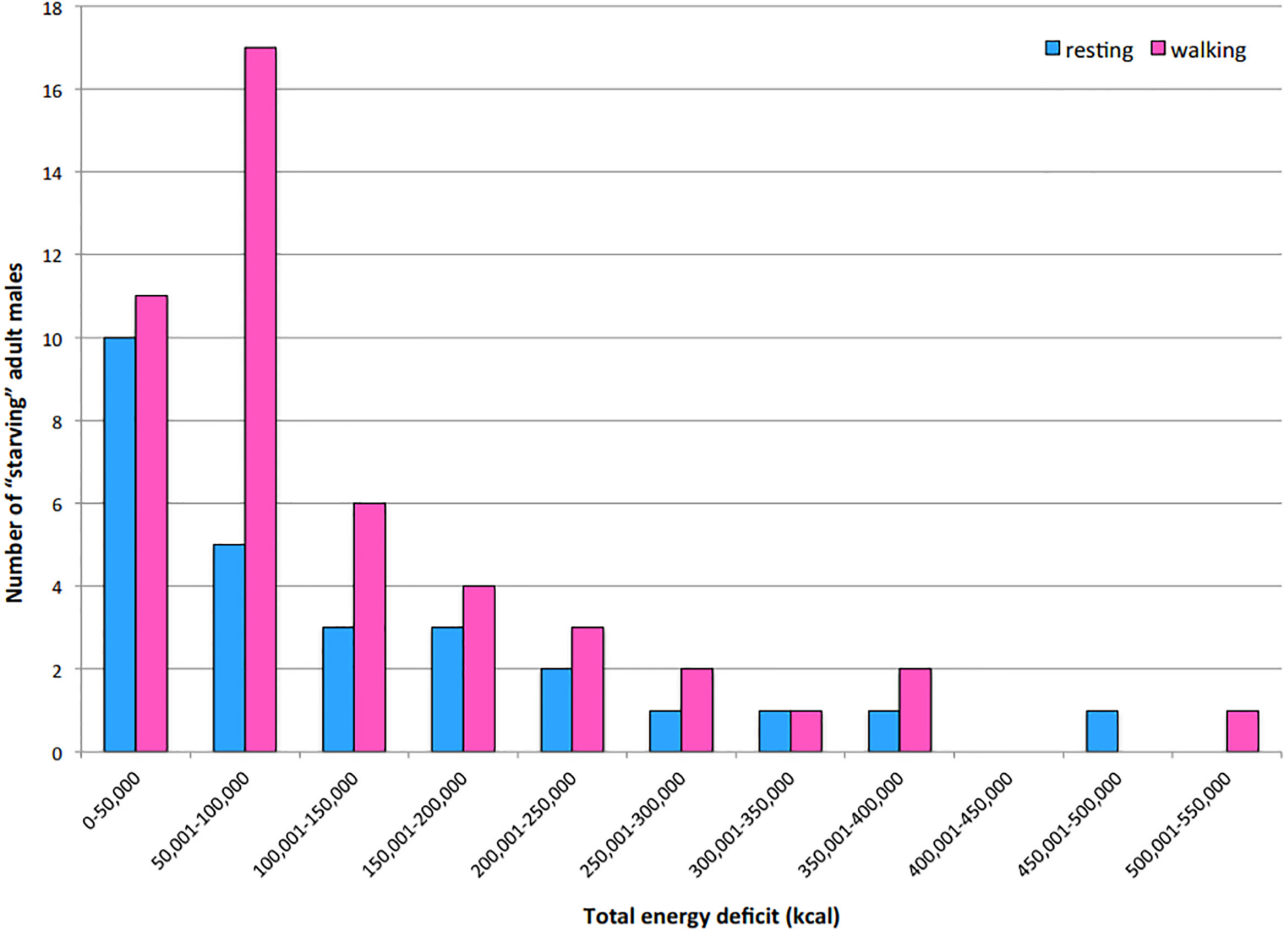
The number of starving adult males that are expected to come ashore with different-sized energy deficits during a 180-day ice-free season. Calculations are based on the additional kilocalories required for daily somatic maintenance and limited movement (2 km d^-1^) needed to prevent starvation in each bear for the entire projected 180 days ashore.

Of the 97 adult male polar bears sampled, Molnár et al. [8] predicted that 28% (27) and 48% (47) would starve if resting or walking before energy depletion, respectively. Of those bears that were resting before starving, we found that many (10) experienced energy deficits of less than 50,000 kcal. Of those bears that were walking before starving, most (28) experienced deficits less than 100,000 kcal, with most falling between 50,000 and 100,000 kcal based on our analyses (Fig 1).

### Potential energy available from land-based foods

**Fig 2:**
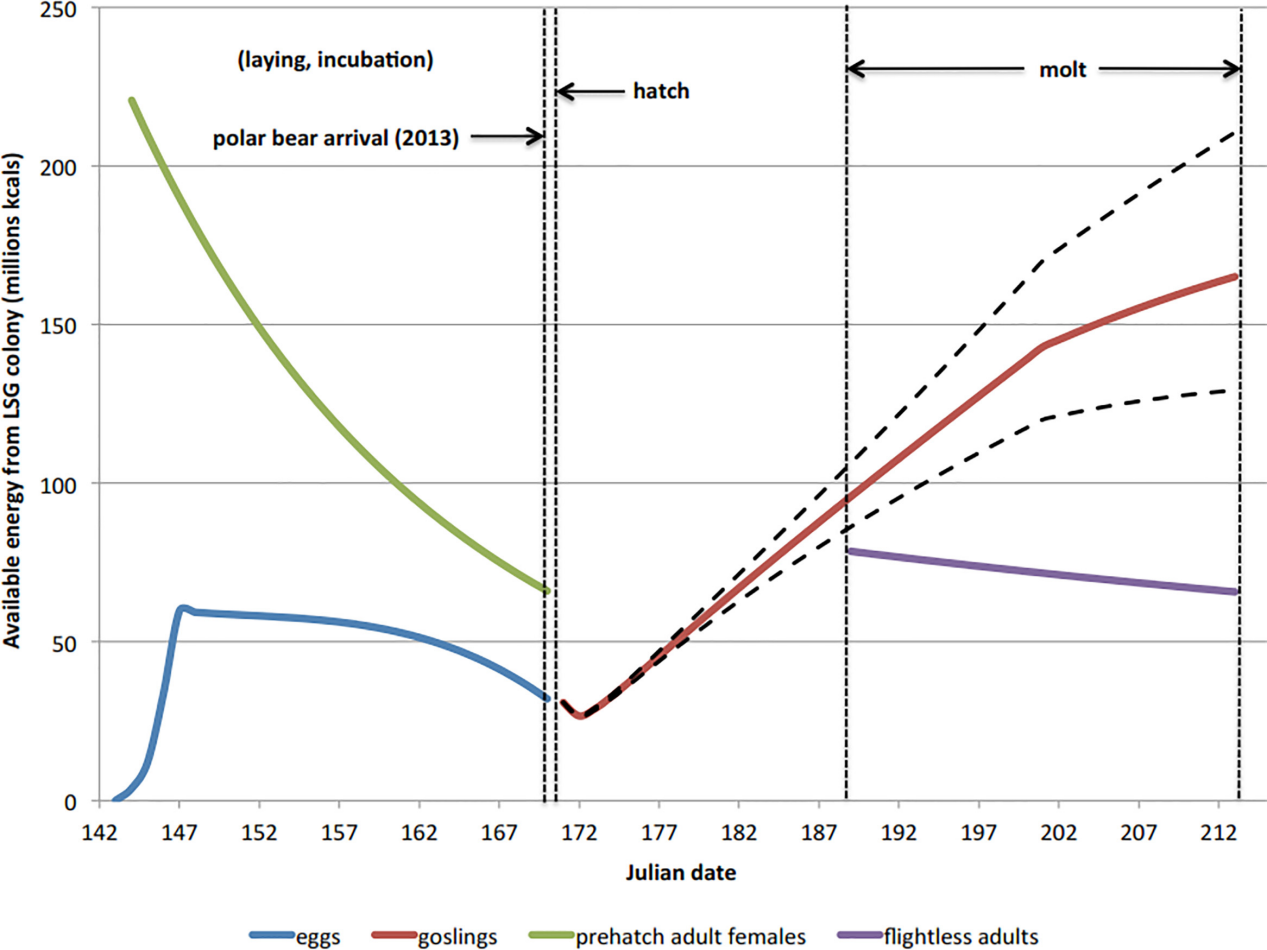
The total available energy from snow geese and their eggs during the laying, incubation, post-hatch and molting stages of their life cycle that occurs in the Cape Churchill Peninsula. The mean hatch and polar bear arrival dates provided (left-most vertical dashed lines) are for 2013, however, if the ice-free season expands to 180 days, polar bears would arrive before nesting geese and thus have access to all the available energy illustrated.

The energetic value of all stages of LSGO (eggs, goslings, adults) calculated for the 2013 population size was approximately 11,702, 10,959 or 10,334 million kcals depending on whether it was a good, average or bad gosling survival year, respectively. During an average gosling survival year, eggs, pre-hatch adult females and flightless adults comprised 11.8%, 31.2% and 16.4% of the total kcals available to polar bears. Goslings comprised 47.4%, 40.6% and 34.9% of the total available kcals in good, average and bad survival years, respectively. The number of clutches and their respective caloric values both dropped over the course of the 24-day incubation period. For example, on day 1 approximately 71,068 clutches were each worth 840.05 kcal, whereas on day 24 the number of clutches drops to 65,027 and were each worth 493.18 kcal. Goslings, available for 43 days, grew rapidly and range in value from 118.68 kcal at hatch to 1128.23 kcal shortly before flight ($\bar{x}$ = 576.61 kcal). Pre-hatch females could provide the most energy per unit and were most valuable during laying and beginning of incubation (3394.46 kcal), then rapidly lost weight over the 27-day period, dropping to 1015.84 kcal just before hatch ($\bar{x}$ = 1950.04 kcal). Flightless adults, having exhausted their fat reserves, could provide between 603.76 kcal (post-hatch) to 505.64 kcal (before flight), with an average value of 552.38 kcal over the 25 days that they were available (Fig 2).

**Fig 3:**
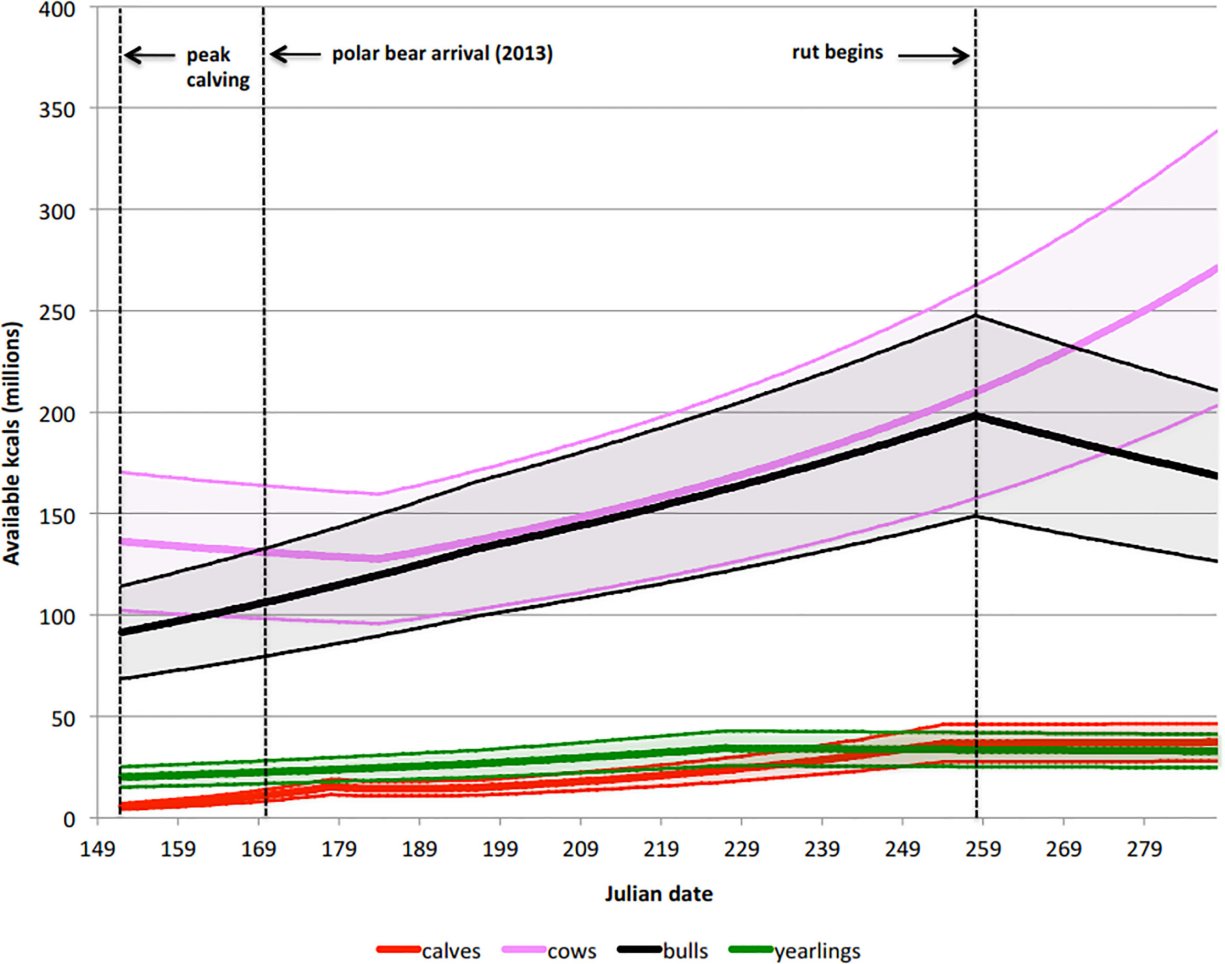
The total available energy from different sex and age classes of caribou on the summer calving grounds on the Cape Churchill Peninsula. If the ice-free season expands to 180 days, polar bears are projected to come ashore prior to the onset of calving, which is currently 1 June, and have access to all the available energy illustrated.

Caribou can provide a total of 38,584, 51,445 or 64,307 million kcal for an estimated population size of 3000, 4000 and 5000, respectively. Assuming an average population size of 4000, calves, yearlings, adult females and adult males comprised 6.2%, 7.8%, 45.9% and 40.1% of the total energy available to polar bears. Adult females were each worth 70,964.09 kcal at the onset of calving and increased to 141,066.20 kcal by the middle of October ($\bar{x}$ = 89,835.99 kcal). Calves and yearlings also steadily gained mass and were each worth 4,751.22 and 34,132.37 kcal, respectively, on 1 June, and increased to 51,653.94 and 55,921.61 kcal by 15 October, averaging 29,539.02 and 50,072.52 kcals, respectively, while on the calving grounds. Individual adult males arrived at the calving grounds potentially worth 64,415.41 kcal and steadily increased in value until the onset of the rut (approx. 15 Sep.), peaking at 139,641.91 kcal, then dropping to 118,835.01 kcal over then next month, averaging 105,956.41 kcal (Fig 3).

### Compensation to starving polar bears

Molnár et al. [8] used the average body lengths (2.34 m) across their sample of adult male polar bears, leaving initial body mass as the sole determinant (except for movement costs) of whether a bear would starve during an extended 180-day ice-free season and for how many days energy compensation would be needed. Assuming the current mean body length has future legitimacy, adult male polar bears (≥ 7 years old) would starve shortly after reaching 191.93 kg and would require approximately 4,450.28 kcal d^-1^ upon reaching that threshold mass to survive.

**Fig 4:**
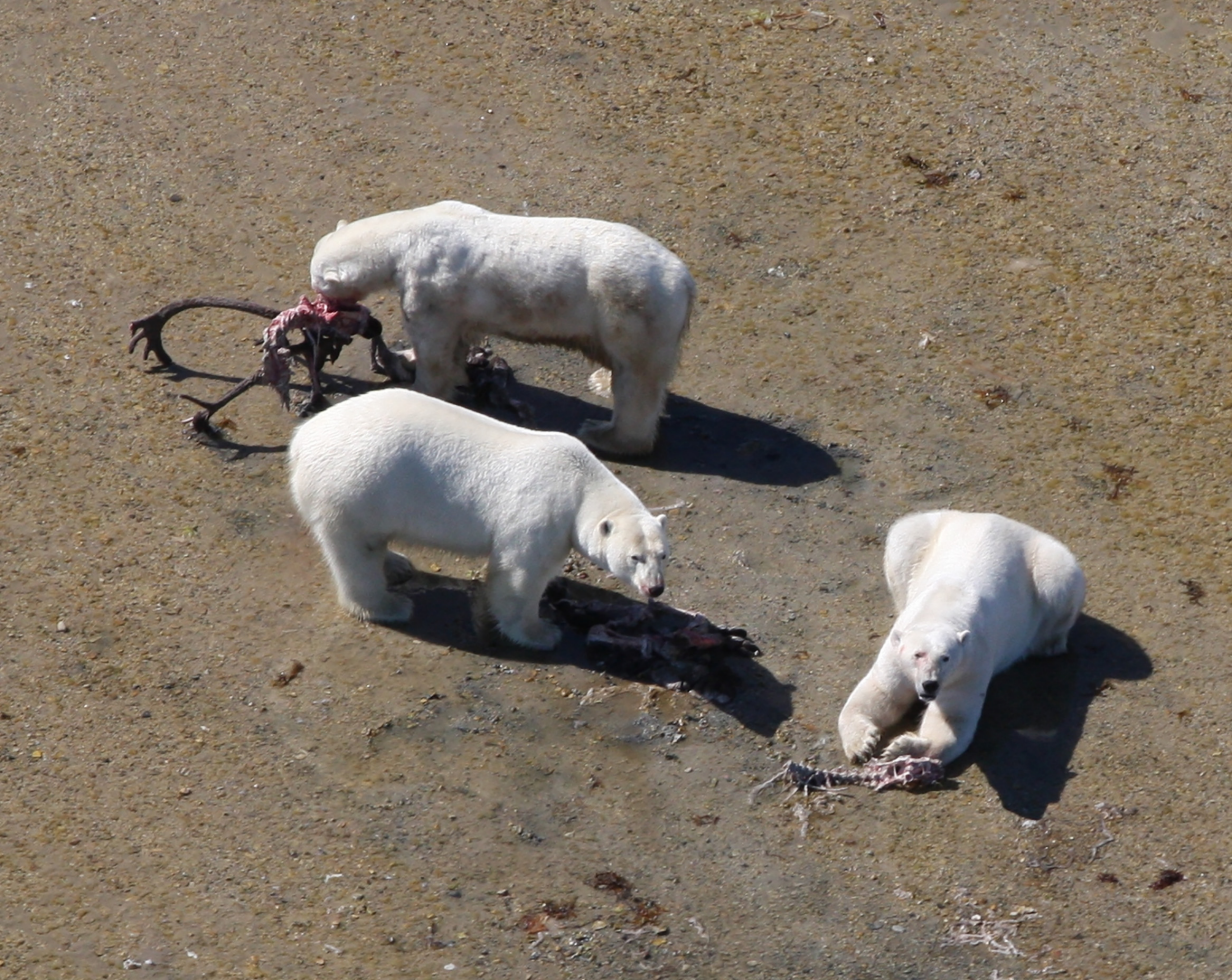
Three adult male polar bears feed on the remains of a bull caribou on Keyask Island (58.1695°N 92.8519°W) on the Cape Churchill Peninsula on 8 August, 2012. This type of communal foraging illustrates the importance of how consumption of incomplete carcasses (as carrion or from predation) can contribute to daily energy requirements. Here, the bear in the poorest physical condition (top) is most likely in need of the additional calories, however, those in better condition still partake in the meal. Photograph by R.F. Rockwell.

**Fig 5:**
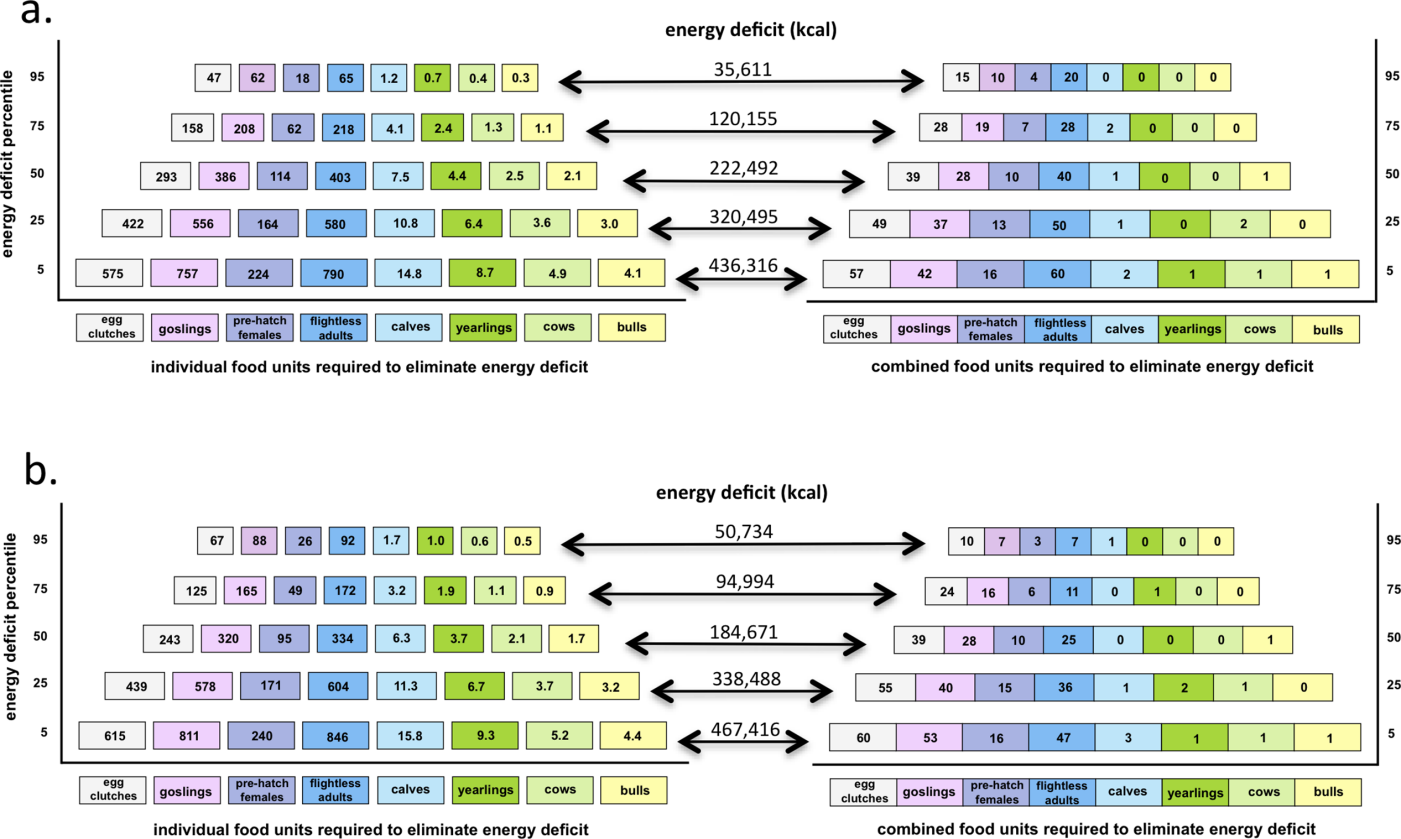
The number of indivudal units (left) or combinations (right) of food items that could satisfy total energy deficits (numbers on arrows in center) of starving male polar bears onshore for 180 days. The levels of energy deficits vary from the lowest (95%) to the highest (5%) and are presented for scenarios in which the bears have been resting (a) or walking (b) on land prior to starving. A 2 km d^-1^ energy cost associated with movement is depreciated from daily food value calculations. If costs to procure food items exceed this movement cost, numbers of individual food requirements may be underestimated.

Adult male caribou could provide the most energy per unit, with less than 5 animals per polar bear (<1 every 27 days) needed to prevent starvation for the entire 180-day ice-free period under scenarios of resting or walking. Because of the high caloric value of each caribou and the incidences of multiple polar bears feeding off a single caribou carcass (Fig 4), the exact proportions of each animal that would be required to meet the daily caloric needs may be important and are presented (Fig 5a and 5b). Calves, though considerably smaller, could still potentially support a starving walking polar bear in the 5^th^ percentile with 15.8 units or approximately 1 calf every 8.7 days while they are available. As expected, smaller food units would require more frequent effort to obtain. For example, to sustain a starving walking bear in the 5^th^ percentile ~26 clutches of eggs or 19 goslings would need to be consumed each day. For starving walking bears in 50^th^ percentile, the effort would drop to 10 clutches and 7.5 goslings per day. The minimum number of units of each food required to sustain polar bears in each condition percentile are presented in Fig 5a and 5b.

Given the opportunistic nature of polar bears [13,21], combinations of food items may be a more realistic means to fulfill daily energy deficits, especially since availability of different age classes of each item does not necessarily overlap (e.g., LSGO, Fig 2). For example, to sustain starving walking polar bears in the 50^th^ percentile would require consuming ~5 egg clutches every 3 days, ~1 incubating female off the nest every 3 days, ~2 goslings every 3 days, 1 flightless adult each day and 1 adult male caribou. Different food combinations for each condition percentile for walking and resting starving polar bears are presented in Fig 5a and 5b.

### Maximum number of starving adult male polar bears supported by each food

**Fig 6:**
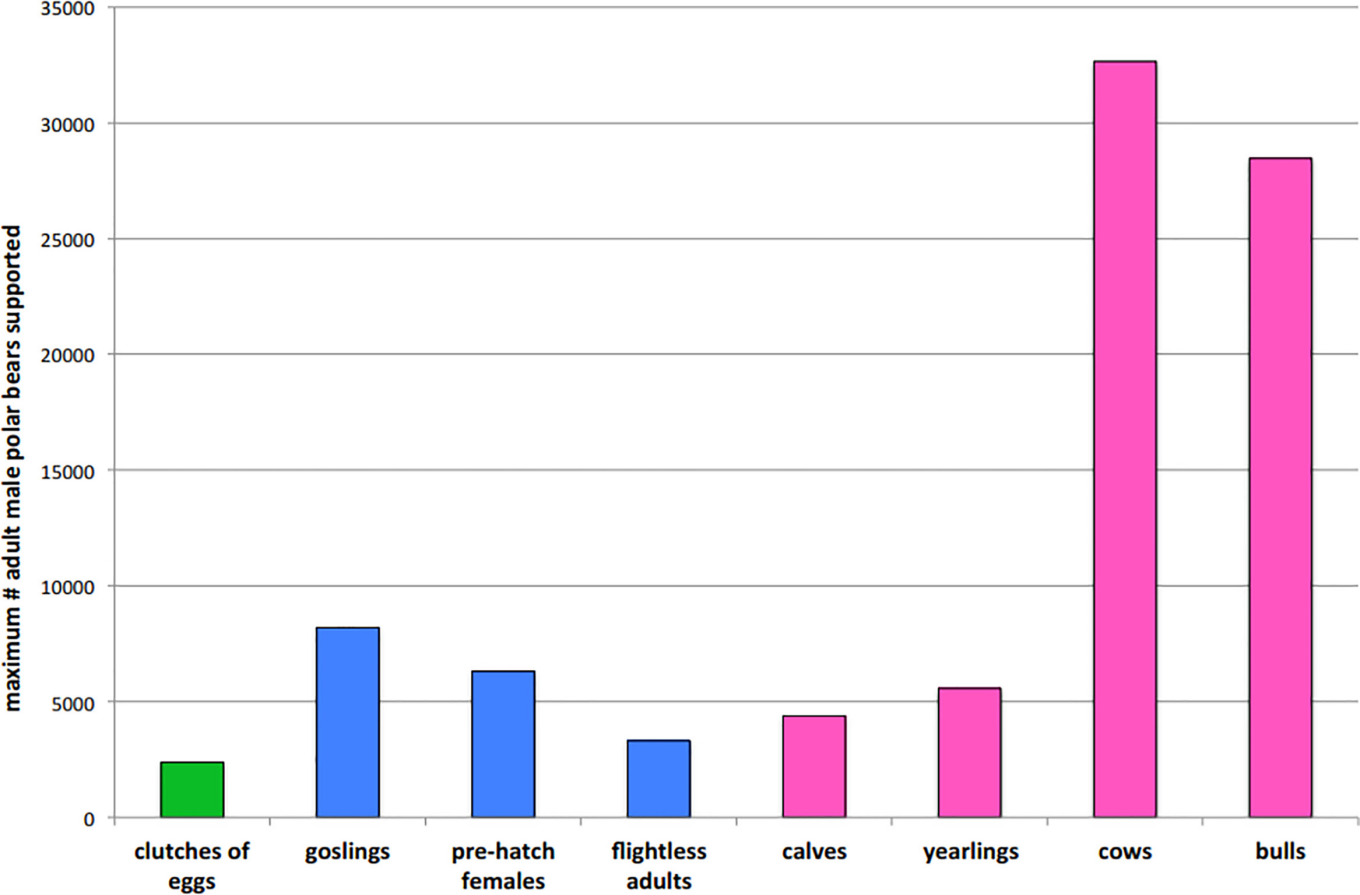
The maximum number of adult male polar bears projected to starve as the ice-free season expands to 180 days that could be supported by the total energy pools from each food resource. Estimates are based on 2013 population sizes of each prey and take into account somatic maintenance and daily movement costs. Values may be overestimated if true procurement costs exceed those included.

Assuming a polar bear population size similar to the last estimate (935) [33] and the proportion of adult males remains constant (~25%), then the available calories from eggs, LSGO and caribou would each far surpass the energetic needs of adult males coming ashore at risk of starvation. To sustain 28% of resting adult males (66 = 935 * 0.25 * 0.28) for 117 days (the maximum # days that a resting starving bear would need supplementation for), the available calories would surpass total energy required to sustain the starving bears by a factor of 38 for eggs to a factor of 520 for adult female caribou. Similarly, to sustain the 48% of walking adult males (112 = 935 * 0.25 * 0.28 * 0.48) that would be susceptible to starvation for 122 days (the maximum time needed), the available calories would surpass those needed by a factor of 21 for eggs to a factor of 291 for adult female caribou. The maximum number of adult male polar bears that could be supported by LSGO for 122 days is 1,614, 5,551, 4,274 and 2,242 by eggs, goslings, pre-hatch adult females and flightless adults, respectively (Fig 6). The maximum number of adult males that could be supported by caribou is 4,378, 5,572, 32,651 and 28,464 by calves, yearlings, cows and bulls, respectively (Fig 6).

## Discussion

As the ice-free season expands with earlier spring breakup, polar bears are expected to come ashore in western Hudson Bay with smaller energy stores [3] causing them to rely on terrestrial food sources to compensate for energy deficits and avoid starvation. Molnár et al. [8] predicted that, depending upon their activity while ashore, between 28 and 48% of adult male polar bears would starve to death unless supplemental food was consumed. By evaluating the energy value of novel foods that polar bears currently consume on land, caribou, snow geese and their eggs, we found that there are sufficient calories to compensate for daily accrued energy deficits of adult male polar bears expected to starve as the ice-free season expands to 180 days. The veracity of relying on these land-based resources, however, likely depends on a number of factors including future prey availability, polar bear foraging behavior, energy costs associated with prey procurement and physiological utilization of different macronutrients.

Although local populations of both snow geese and caribou have grown substantially since the 1960s [13], future availability is difficult to predict. Both species have displayed weak phenological shifts in response to climate change, as reproductive cycles are cued more by day length than air temperature [34,35]. As a result, mismatches with emergent vegetation at the peak of goose brood rearing and peak caribou calving as well as habitat changes have impacted both species [57,67]. Snow geese in the CCP have responded to changes in food availability (mostly due to destructive foraging) by inhabiting new areas, moving further inland from the coast and consuming alternative plant species [67]. Further, there is evidence to suggest that deficiencies from mismatches with emergent vegetation at the onset of brood rearing may, in part, be compensated for by earlier access to berries later in the season (C.P. Mulder, unpublished data). Behavioral changes, such as range shifts, are possible with heavy predation, however, the lack of such responses from other nesting geese experiencing arctic fox and polar bear predation suggests that local snow goose populations would likely not alter their behavior substantially (see [26] and references therein). Such adaptive responses to environmental stresses and resilience in the face of rigorous management control attempts [38,68] suggests that snow geese may remain a viable future food source for polar bears on the CCP.

The resiliency of caribou in the face of progressive environmental change is less certain [57]. Although the Churchill herd is currently stable, studies in other regions have suggested that increases in variability and advances in emergence of commonly consumed plants with warming temperatures have negatively impacted calf survival [35]. Other threats have included replacement of preferred winter forage (i.e., lichen and herbaceous plants) with shrubs and grasses from forest fires, grazing and warmer temperatures [69,70,71]. Also, projected increases in precipitation would give predators, such as wolves, an advantage potentially increasing mortality [72]. These changes, however, can affect populations adapted to harsh conditions in different ways [73], so that some populations are experiencing growth while others decline [57]. Given the small number of animals required to satisfy the energetic requirements of starving polar bears, it is unclear whether even modest future declines in the Churchill herd would hinder polar bear predation efforts as long as caribou maintain their current distribution (i.e., along the coast). Caribou may occupy the coast of western Hudson Bay (where polar bears occur in high densities) for a variety of reasons including to avoid harassment by insects (e.g., [74]) so whether the increased threat of predation would cause them to shift their distribution further inland is unknown. Encounter rates between polar bears and caribou may decrease with declines in abundance and shifts in distribution, but the method of capture (e.g., ambush versus chase) may be more important in determining predation success [75,76].

With the paucity of knowledge regarding energy consumption rates at varying speeds of travel, especially for adult males (i.e., > 235 kg), it is difficult to fully evaluate the feasibility of the foraging scenarios suggested. However, based on past and current behavior, it is clear that polar bears are capable of successfully capturing land-based ungulates, such as caribou (R.F. Rockwell, pers. obs., [77]) and muskoxen [78] and actively pursuing them in western Hudson Bay and other regions (L.J. Gormezano, pers. obs., [23,56]). The mean digestible energy content of a seal, pooled across age classes, is 69,047 kcal [79], which is roughly equivalent to the average worth of an adult female caribou during June and July, although most of the energy is from protein rather than fat. Using surprise hunting techniques, such as stalking and ambushing, whereby landscape features (i.e., ice, water) are used to mask their approach, polar bears are able to successfully capture seals without engaging in potentially costly pursuits [80,81]. Polar bears have employed these same techniques on land, using shrubs and physiographic features as cover to surprise caribou (R.F. Rockwell, pers. obs., [56]) suggesting equivalent (seal) calories could be obtained on land without drastic changes in energy output. Further, caribou capture rates (1 every 8.7–31.1 days) required to sustain starving walking polar bears coming ashore in the worst condition, are comparable to capture rates (1 seal every 5.6–24.4 days) [4] of different aged seals by polar bears in spring and summer.

Consumption rates of snow goose eggs and goslings that would compensate starving walking bears ranged from 3 to 26 clutches of eggs and 2 to 19 goslings per day depending on daily energy deficits. High daily depredation rates of goose nests have been reported in populations around the Arctic (e.g., 108 barnacle goose nests) [82] but can vary depending on nest density and total availability [83,84]. Based on camera footage of an common eider colony in western Hudson Bay, some polar bears were consuming between 19 and 38 nests per day (D. Iles, unpublished data) suggesting that the maximum consumption rates required to support starving bears (26 clutches) is not unrealistic. Also, polar bears coming ashore 60 days earlier (as would be projected for 2068), they would overlap the entire incubation period, which would provide substantially more calories to arriving bears than are currently available [34].

Capture rates of goslings and adults are rarely reported [85,86] but observations of polar bears capturing and consuming up to 3 individuals per day (of various ages) have been reported for snow geese [22]. The extra skill and effort required to obtain birds (as opposed to eggs) might pose limitations on meeting daily energy requirements from birds alone, however, maximum rates suggested in the combination diet (0.2 to 1.2 goslings d^-1^, for example) are quite reasonable. Further, anecdotal observations of flightless goslings and adult snow geese being consumed consecutively by the same bear [22] and remains of adult snow geese and eggs recorded in the same scat (25% of scats with eggs) further suggests a combination diet (Fig 5a and 5b) would be a more realistic means to satisfy daily energy requirements.

The large numbers of starving polar bears that can be supported by each food resource suggest that surpluses would be available for other age and sex classes coming ashore with energy deficits. It is important to note, however, that the actual number of bears that will come ashore with energy deficits is unknown and may increase over time as marine food resources become limited. Further, the only costs associated with procurement of prey are a 2 km d^-1^ movement cost above somatic maintenance. Similar to lions (*Panthera leo)*, polar bears are considered inefficient walkers so extended pursuits could reduce energetic returns [87]. Pursuits of geese on land rarely exceed 30 seconds (R.F. Rockwell, pers. obs., [22]), however, pursuits of caribou (running, walking and swimming) have lasted up to an hour (L.J. Gormezano, pers. obs., [23,56]) suggesting that costs associated with each capture (including failed attempts) could be substantial.

Williams and Yeates [88] calculated an efficiency ratio (benefits/costs) of 3.8 for African lions pursuing ungulates on land. Given the comparable locomotive inefficiencies between lions and polar bears (Gormezano and Rockwell, unpublished data, [87,89]) it is possible that when polar bears engage in longer distance pursuits, as opposed to more energy conserving surprise techniques, a similar efficiency ratio could apply. In a hypothetical example, we applied this ratio to the energetic returns for caribou and found that it increased capture costs (i.e., above somatic and movement) 1.7, 3.0, 5.3 and 6.3 times their previous value of approximately 4,450 kcal for calves, yearlings, adult females and adult males, respectively. Applying these increased costs to the calculation of the number of starving walking polar bears supported by the total calories from adult male caribou, for example, would reduce the number supported by 84% from 28,464 to 4,543 bears. Although the exact energetic costs of polar bears pursuing caribou using different hunting strategies remain unknown, the data presented here provide a basis to estimate them once the appropriate behavioral and energetic studies have been performed.

Previous studies have questioned the use of land-based foods to satisfy daily energy requirements while polar bears are on land [90,91]. Hobson et al. [11], for example, tested carbon dioxide exhaled by anesthetized polar bears in summer to evaluate whether a marine (seal) or terrestrial (berry) stable-carbon signature would be obtained and thus, which was supplying energy for current metabolic processes. Finding signatures that were almost identical to seals (and different from berries), they concluded that all bears were persisting solely on energy derived from oxidized fat reserves accumulated while on the ice [11].

It is possible, however, that the metabolic states of the bears in the Hobson et al. [11] study were altered due to the biochemical effects of being captured [92]. Using the same drugs and capture protocol, Cattet [12] found that the plasma cortisol levels of polar bears after capture were extremely elevated and although they decreased 40–50% after 1 hour, he noted that the physiological effects would continue well after the plasma cortisol levels decreased. He also observed sustained concentrations of plasma glucose correlated with the cortisol surge and suggested the bears may be exhibiting insulin resistance [12]. One of the many effects of cortisol is to sensitize adipose tissue to the action of lipolytic hormones and to cause insulin resistance by decreasing the rate at which insulin activates the glucose uptake system [93]. As a result, insulin resistance leads to the disinhibition of lipolysis in humans [94]. If similar processes occur in polar bears, the use of fat as a metabolic fuel that Hobson et al. [11] observed may not represent the prevalent process, but instead, may have been temporary and triggered by the stress of capture [92].

Furthermore, certain foods that polar bears consume on land can complicate results of biochemical studies to distinguish ‘marine’ versus ‘terrestrial’ sources of expended energy using stable carbon isotopes (δ13C) [14,95,96]. For example, marine algae (*Laminaria* spp. and *Fucus* spp.), typical C4 plants that polar bears commonly consume from land, are more enriched with carbon and have higher δ13C values (-24 to -12‰) compared to most C3 (terrestrial) plants, although values range widely depending on plant part and time period sampled [14,95].

Similarly, waterfowl, such as snow geese, Canada geese (*Branta canadensis*) and common eiders, summering on land in western Hudson Bay can exhibit ‘marine’ signatures from foraging on plants and animals in brackish marshes and marine habitats [97,98]. Muscle δ13C values for the aforementioned and other seabirds that polar bears consume can range from (-22.0 to -15.5‰) [15,97,98], which clearly overlap δ13C values for ringed seal muscle (-19.4 to -18.1‰) [90,97] and could, therefore, lead to erroneous conclusions regarding the sources of energy used on land. Without specifically including these terrestrial foods (i.e., marine algae, birds) a priori in carbon isotope mixing models, their proportional contribution can not be accurately assessed [99], especially given the range of food combinations observed in the summer diet from scat analysis [21].

Earlier arriving bears may come ashore with greater nutritional deficits from lost seal hunting opportunities as the ice-free season expands [7] but calories necessary to prevent starvation will likely be available from land-based resources, such as caribou, snow geese and eggs. The projected earlier 60-day arrival would allow polar bears to overlap both the entire incubation and calving periods of snow geese and caribou, respectively, creating new phenological matches to compensate for the growing mismatch with seals. Using the same energy-saving, surprise hunting methods (e.g., ambush, stalk) to hunt geese and caribou that they typically use to capture seals [80,81], would provide polar bears energy compensation similar to the maximum values reported here. Until further behavioral and oxygen consumption studies are performed, however, the true costs associated with different foraging strategies and thus the total energy returns can only be approximated.
